# A review of tigecycline resistance in nontuberculous mycobacteria

**DOI:** 10.1007/s11033-026-12747-1

**Published:** 2026-09-27

**Authors:** Camila da Silva Moura, William Marco Vicente da Silva, Jesus Pais Ramos

**Affiliations:** Laboratório de Referência Nacional de Tuberculose e Micobacterioses, Centro de Referência Professor Hélio Fraga, Escola Nacional de Saúde Pública, Fiocruz, RJ Brasil

**Keywords:** Antimicrobial, Mycobacterium, Nontuberculous mycobacteria, Resistance, Tigecycline

## Abstract

The genus *Mycobacterium* comprises nontuberculous mycobacteria (NTM), which are opportunistic environmental pathogens primarily associated with chronic lung disease. Among current therapeutic options, tigecycline has emerged as a promising alternative for treating NTM due to its broad spectrum of activity, including efficacy against certain mycobacterial species. In this review, we compiled 28 studies investigating the mechanisms and prevalence of tigecycline resistance in NTM. The evidence suggests that tigecycline is less impacted by the monooxygenase MabTetX, the principal tetracycline resistance determinant in *M. abscessus*. However, adaptive mechanisms, such as WhiB7 regulation, alterations in the *sigH–rshA* axis, and overexpression of efflux pumps (MmpL), can increase the minimum inhibitory concentration (MIC). In contrast, slow-growing NTM species, including *M. avium* and *M. kansasii*, exhibit low intrinsic susceptibility, reflected by high MIC values. We conclude that tigecycline is a promising component of combination of regimens for rapid-growing NTM, although its efficacy against slow-growing species remains limited and standardized breakpoints have not yet been established. .

**Supplementary Information:**

The online version contains supplementary material available at https://doi.org/10.1007/s11033-026-12747-1.

## Introduction

### Mycobacteria

Comprising a large number of species, the genus *Mycobacterium* includes well-known pathogens, such as the *Mycobacterium tuberculosis* complex, which causes tuberculosis [1], *M. leprae*, the etiological agent of leprosy [2], and nontuberculous mycobacteria (NTM). NTMs are ubiquitous in the environment and can cause opportunistic infections in humans, especially in immunocompromised individuals [3, 4]. They are predominantly responsible for pulmonary disease in patients with pre-existing respiratory conditions, but also include skin and soft tissue infections, lymphadenitis, and disseminated forms of the disease [5].

The capacity of NTM to form biofilms facilitates colonization of medical devices and water distribution systems [6]. Transmission primarily occurs through inhalation of contaminated aerosols, particularly in humid environments such as showers, hot tubs, thermal springs, and ventilation systems [7]. Infection can also result from inoculation during invasive procedures, including surgeries, injections, and the use of contaminated medical instruments. Additionally, infections have been reported following aesthetic procedures, such as tattoos, piercings, and skin cleaning [6]. Person-to-person transmission is uncommon with most infections acquired from environmental sources. However, evidence indicates potential transmission between individuals in populations such as cystic fibrosis patients or in specific healthcare settings [8].

Currently, the World Health Organization does not issue a global prevalence report for nontuberculous mycobacteria (NTM). As a result, global estimates of NTM prevalence are derived from national cohort studies and systematic literature reviews, rather than official WHO statistics. Recent investigations of this nature have consistently shown an increase in both the incidence and prevalence of NTM over recent decades, with a particularly pronounced effect in low-income nations [9–11]. Moreover, available evidence regarding pulmonary NTM disease and culture-positive isolates indicates that *M. avium* complex (MAC) remains the predominant species across the majority of countries [9].

### Tigecycline and bacterial resistance

The management of NTM infections remains challenging because mycobacteria possess high intrinsic resistance to numerous antimicrobials [12]. Tigecycline is a member of the glycylcycline antibiotic class and a derivative of minocycline. This antibiotic exhibits a broad-spectrum antimicrobial activity against Gram-positive and Gram-negative bacteria as well as certain mycobacteria. Its mechanism of action involves binding to the 30 S subunit of the bacterial ribosome, thereby inhibiting the entry of aminoacyl-tRNA into the A site, consequently blocking bacterial protein synthesis [13, 14].

Multiple resistance mechanisms contribute to the reduced susceptibility of NTM to tigecycline and other antimicrobial agents. The lipid-rich mycobacterial cell wall, characterized by abundant mycolic acids and a low density of porin channels, acts as an important permeability barrier that limits antibiotic penetration [15]. Efflux pumps constitute another major resistance mechanism by actively exporting antimicrobial compounds from the bacterial cell. In *M. abscessus*, overexpression of efflux pump systems has been associated with reduced susceptibility to tigecycline and other ribosome-targeting antibiotics [15]. Additionally, alterations in ribosomal proteins and ribosomal RNA may reduce tigecycline binding, although these mechanisms remain incompletely characterized in NTM. The considerable variability in tigecycline susceptibility observed among *M. abscessus* isolates suggests that genetic determinants play an important role in modulating antimicrobial activity [16]. Biofilm formation further contributes to phenotypic resistance by reducing antibiotic penetration and promoting bacterial persistence [17].

Overall, the available evidence supports tigecycline as an important component of combination therapy for infections caused by rapidly growing NTM, particularly multidrug-resistant isolates. Characterizing tigecycline resistance patterns and understanding the molecular mechanisms involved are essential for optimizing antimicrobial therapy, improving clinical outcomes, and providing scientific evidence to support the establishment of standardized susceptibility breakpoints and the rational clinical use of tigecycline.

### Clinical manifestations

The clinical manifestations of nontuberculous mycobacterial (NTM) infections vary according to the infecting species, the host’s immune status, and underlying comorbidities. Pulmonary disease is the most common presentation, particularly in patients with structural lung diseases such as bronchiectasis, chronic obstructive pulmonary disease, cystic fibrosis, and a history of tuberculosis. Typical symptoms include chronic cough, sputum production, fatigue, dyspnea, weight loss, low-grade fever, and hemoptysis, a clinical picture that often resembles pulmonary tuberculosis and may delay diagnosis [18, 19].

Extrapulmonary disease is less frequent and occurs predominantly in immunocompromised individuals. Common manifestations include skin and soft tissue infections following trauma or invasive procedures, cervical lymphadenitis, and disseminated disease involving multiple organs, particularly in patients with advanced immunosuppression [18, 20]. The broad clinical spectrum of NTM infections highlights the importance of accurate species identification and antimicrobial susceptibility testing to guide appropriate therapy and improve clinical outcomes [19].

## Materials and methods

This review was conducted using predefined search strategies across the following electronic databases: Scopus, Public MEDLINE **(**PubMed), Virtual Health Library (VHL), Scientific Electronic Library Online (SciELO), Web of Science and Latin American and Caribbean Health Sciences Literature (LILACS). The search utilized the following keywords: *Mycobacterium*, mycobacteria, nontuberculous mycobacteria, non-tuberculous mycobacteria, NTM, MNT, tigecycline, tigeciclina, resistance, resistência, susceptibility, suscetibilidade, minimum inhibitory concentration, MIC. The Boolean operators OR and AND were used to combine the terms. Additionally, a language filter was applied to include only publications in English and Portuguese. The searches and the study selection process were conducted between September and December 2025.

After implementing the search strategy, articles were manually screened to identify those suitable for inclusion in the present study. The inclusion criteria comprised studies addressing resistance of nontuberculous mycobacteria to tigecycline, published in English or Portuguese language peer-reviewed journals, with no restriction on publication year. Duplicate articles were removed.

## Results

An initial search using specific descriptors identified 42 potentially relevant articles. Following a review of titles and abstracts, some studies that did not meet the pre-established inclusion criteria were excluded. Subsequently, 28 articles were selected for full-text review and included in the analysis (Supplementary Table S1). The majority of these studies reported an association between nontuberculous mycobacteria (NTM) and tigecycline in their titles or abstracts, with *M. abscessus* identified as the most frequently encountered species [21].

Guo et al. [21] reported that tigecycline exhibits strong in vitro activity against the *M. abscessus* complex (MABC), including *M. abscessus* and *M. massiliense*, as indicated by consistently low MIC values. Similarly, Wallace et al. [22] found high susceptibility to tigecycline among rapid-growing mycobacteria (RGM), including *M. abscessus* (MIC₅₀ ≤ 0.12 µg/mL; MIC₉₀ = 0.25 µg/mL), *M. chelonae*, and *M. fortuitum*, all of which demonstrated comparably low MIC values (≤ 0.12 µg/mL).

Recent studies have demonstrated heterogeneity within this profile. Ng et al. [24] identified strains exhibiting resistance or reduced susceptibility to tigecycline, despite the initially promising in vitro activity of the drug. Similarly, Brown-Elliott et al. [16] observed phenotypic differences among clinical isolates.

Rudra et al. [24] and Singh et al. [25] reported that intrinsic resistance to classical tetracyclines in *M. abscessus* is associated with the monooxygenase enzyme MabTetX, encoded by *MAB_1496c*. They also observed that expression of *MAB_1496c* increases in the presence of tetracycline and doxycycline, whereas tigecycline does not induce its expression and remains more stable against MabTetX-mediated inactivation.

Hurst-Hess et al. [26] and Singh et al. [25] reported that deletion of *whiB7* increased susceptibility to tigecycline, whereas expression of *whiB7* was associated with increased MIC. Hurst-Hess et al. [27] further identified *whiB7* as a transcriptional activator of *erm41*, *hflX*, *eis2* and *ABCF*-family genes.

Ng et al. [28] reported a spontaneous *M. abscessus* mutant (7 C), derived from the ATCC 19977 strain, that exhibited tigecycline resistance, cross-resistance to imipenem, and a modest reduction in growth rate. Genomic analysis revealed a mutation in the *MAB_3542c* gene, (homologous to *rshA*). Subsequent studies [28, 29] demonstrated decreased expression of ribosomal genes and transient resistance induced by heat stress.

Aw et al. [30] examined the genetic mechanisms underlying tigecycline resistance in *M. abscessus*, with particular emphasis on mutations in the *rshA* gene, which encodes anti-sigma factor regulating the sigma factor SigH. Analysis of two mutant strains, CL5A and CL6A, exhibiting resistance or decreased susceptibility to tigecycline, revealed that mutations in *rshA* disrupt the interaction between RshA and SigH, leading to SigH overexpression. This alteration in bacterial stress-response regulation was associated with tigecycline resistance, indicating that regulatory mechanisms, in addition to antibiotic-modifying enzymes, can reduce efficacy of tigecycline in *M. abscessus* [30].

A further mechanism identified involves the overexpression of efflux pumps in the MmpL family, particularly MmpL5, which is associated with reduced intracellular antibiotic concentration. Mudde et al. [31] demonstrated that 24-hours exposure to tigecycline significantly induces the expression of these efflux systems. The principal genetic and molecular mechanisms associated with tigecycline resistance in NTM are synthesized in Table 1.

**Table 1:** Genetic mutations, molecular mechanisms, specific study outcomes, and interpretation associated with tigecycline resistance in nontuberculous mycobacteria (NTM)

NTM species / RGM	Mutations / Alterations	Resistance mechanism	Number of resistant strains	Specific outcomes	Interpretation in this review	Related articles
*M. abscessus*	Gene *MAB_1496c* (MabTetX) – not induced by tigecycline	Enzymatic inactivation of classical tetracyclines (monooxygenase); tigecycline is a poor substrate	Not specified	Tigecycline remained active despite TetX homolog; no relevant MIC increase observed	Tigecycline bypasses classical tetracycline resistance mechanisms, supporting its clinical utility	[24, 26, 27]
*M. abscessus*	Deletion or mutation in *whiB7*	Global transcriptional regulator; induction of resistance genes (e.g., efflux pumps)	Mutant strain	Altered expression of intrinsic resistance genes; variable impact on tigecycline susceptibility	Suggests indirect modulation rather than a direct resistance mechanism	[23, 25, 26, 33]
*M. abscessus*	C51R mutation in *rshA* / mutations in *sigH–rshA*	Dysregulation of stress response; SigH overexpression; reduced ribosomal gene expression	3 strains (CL5A, CL6A, variant 7 C)	Increased tolerance to tigecycline linked to stress-response activation	Indicates adaptive resistance and persistence mechanisms rather than stable resistance	[23, 27, 29, 31]
*M. abscessus*	MmpL5 efflux pump overexpression	Reduced intracellular antibiotic concentration	Not specified	Decreased susceptibility associated with increased efflux activity	Efflux plays a central role; potential target for adjunctive therapies	[16, 26, 30]
*M. chelonae / M. fortuitum*	Not related	Not specified	76 isolates (all susceptible)	Uniform susceptibility to tigecycline observed	Reinforces tigecycline effectiveness against these rapidly growing mycobacteria	[22]
MAC, *M. kansasii*, *M. marinum*, *M. gordonae*, *M. simiae*, *M. szulgai*, *M. xenopi*, *M. scrofulaceum*, *M. malmoense*	Not related	Not specified	45 isolates (all resistant; species not determined)	High resistance reported, but species-level data lacking	Limits conclusions; suggests possible intrinsic resistance among slow-growing NTM	[22]
*M. avium / M. intracellulare / M. kansasii*	Not related	Intrinsic resistance (cell wall barrier, slow growth, basal efflux)	Not specified	Reduced susceptibility attributed to intrinsic physiological traits	Resistance is likely intrinsic rather than mutation-driven	[22, 23, 31]

Ng & Ngeow [23] reported that other rapidly growing nontuberculous mycobacteria such as *M. chelonae* and *M. fortuitum* also exhibit a favorable susceptibility to tigecycline. Nevertheless, information regarding the underlying molecular mechanisms remains scarce.

In contrast, slow-growing mycobacteria showed low susceptibility to tigecycline. Wallace et al. [22] observed consistent resistance with MIC exceeding 4 µg/mL. Li et al. [32] reported MIC₅₀/MIC₉₀ values of at least 8 µg/mL for *M. avium*, *M. intracellulare*, and *M. kansasii*.

Daley et al. [33], in a systematic review of 89 studies, confirmed that tigecycline demonstrates greater activity against rapidly growing NTM, whereas evidence supporting its efficacy in slow-growing species remains limited.

Schildkraut et al. [34] reported that tigecycline exposure induces transcriptomic responses in *M. abscessus*, including increased expression of *sigH*. Induction of WhiB7 was also observed. Hurst-Hess et al. [27] demonstrated that deletion of *whiB7* increased susceptibility to tigecycline, while deletion of *sigH* resulted in a modest increase in susceptibility. Overexpression of *MAB_4664* was associated with reduced susceptibility. These findings collectively suggest that the transcriptional response triggered by tigecycline is functionally associated with mechanisms that modulate antibiotic susceptibility in *M. abscessus*.

## Discussion

### Overall activity of tigecycline against nontuberculous mycobacteria

The findings of this review indicate that tigecycline exhibits significant in vitro activity against NTM, particularly against rapidly growing species such as *M. abscessus*, *M. chelonae*, and *M. fortuitum*. Early investigations, including those by Wallace et al. [22] and Guo et al. [21], reported low MIC values, suggesting a relatively consistent spectrum of activity among these species. However, more recent studies [16, 23] have demonstrated that susceptibility is not uniform, particularly in *M. abscessus* complex, where increased phenotypic variability and the emergence of isolates with reduced susceptibility have been documented.

Although most studies consistently report favorable in vitro activity of tigecycline against rapidly growing mycobacteria, direct comparison among studies remains challenging because of substantial methodological heterogeneity. Differences in broth microdilution protocols, culture media, incubation periods, isolate selection criteria, and sample size likely contribute to the variability in reported MIC values. Moreover, isolates were obtained from different geographic regions, suggesting that regional genetic diversity may also influence susceptibility profiles. Consequently, the observed variation in MIC values is probably multifactorial and cannot be attributed exclusively to resistance mechanisms.

The variability in tigecycline susceptibility likely reflects the combined influence of intrinsic bacterial characteristics and adaptive responses. Baseline differences in regulatory gene expression, activation of stress-response pathways, induction of efflux systems, and genetic diversity among clinical isolates may all contribute to differences in antimicrobial susceptibility. These biological factors, together with methodological variability between susceptibility assays, probably explain much of the heterogeneity observed across published studies.

Despite physiological similarities among rapidly growing mycobacteria, important interspecies differences exist in their ability to activate adaptive responses under antibiotic exposure. In contrast, slow-growing species, including *M. avium*, *M. intracellulare*, and *M. kansasii*, consistently exhibit higher MIC values, indicating lower susceptibility to tigecycline [22, 32]. In these organisms, reduced susceptibility appears to be predominantly associated with intrinsic characteristics, including the highly lipid-rich, poorly permeable cell envelope and lower metabolic activity, rather than the adaptive regulatory mechanisms that have been extensively characterized in *M. abscessus*. Consequently, current evidence supports the greater therapeutic potential of tigecycline against rapidly growing NTM, whereas its role in slow-growing species remains limited.

### Molecular mechanisms underlying reduced susceptibility to tigecycline

Current evidence indicates that reduced susceptibility to tigecycline in NTM is a multifactorial process involving intrinsic structural barriers and complex adaptive regulatory responses rather than mutations affecting a single molecular target. Unlike many bacterial pathogens in which resistance primarily results from target-site modifications, tigecycline resistance in NTM appears to arise through coordinated physiological adaptations that collectively reduce intracellular drug activity.

Intrinsic low permeability of the mycobacterial cell envelope constitutes the first barrier limiting tigecycline activity. The complex cell wall, composed of mycolic acids, arabinogalactan, peptidoglycan, and diverse glycolipids, together with the limited number of porins, restricts antibiotic penetration. This intrinsic characteristic is particularly relevant in slow-growing species and contributes to their naturally higher MIC values. However, cell wall impermeability alone does not fully explain the variability in susceptibility observed among rapidly growing mycobacteria.

Among the transcriptional regulators associated with tigecycline susceptibility, WhiB7 is considered the central regulator of the intrinsic antibiotic stress response in mycobacteria. This transcription factor functions as a master regulator by inducing the expression of multiple resistance-associated genes, including *erm(41)*, *eis2*, *hflX*, and members of the *ABCF* family, thereby promoting adaptive resistance to several antimicrobial classes [27, 35, 36]. Experimental deletion of *whiB7* significantly increases susceptibility to tigecycline, whereas activation of this regulator promotes adaptive resistance [25, 26]. Recent transcriptomic studies further demonstrate that WhiB7 operates hierarchically, integrating additional regulatory pathways, including those involving the alternative sigma factor SigH, thereby coordinating a broad bacterial response to antibiotic-induced stress. Although the role of WhiB7 in tigecycline resistance has gained increasing attention, the regulatory mechanisms governing this phenotype remain an active area of investigation.

Another transcriptional regulator implicated in tetracycline susceptibility is MabTetRx, a TetR-family transcriptional repressor that negatively regulates the expression of *MAB_1496c*, which encodes the flavin-dependent monooxygenase MabTetX. Rudra et al. [24] demonstrated that intrinsic resistance to classical tetracyclines in *M. abscessus* is mediated by MabTetX. In contrast, tigecycline exhibits low affinity for this enzyme and does not induce its expression, thereby maintaining activity against isolates resistant to older tetracyclines [24, 25]. Although MabTetRx has not been directly associated with tigecycline resistance, its regulatory role within the *MabTetX* operon suggests that it may contribute to adaptive responses involved in the evolution of reduced tigecycline susceptibility. Nevertheless, this structural advantage does not prevent the emergence of resistance mediated through alternative adaptive pathways [24].

Among these alternative regulatory pathways, the SigH-RshA regulatory axis has also been associated with reduced tigecycline susceptibility. SigH, an alternative sigma factor involved in the mycobacterial stress response, becomes constitutively activated by mutations in *rshA*, leading to extensive transcriptional reprogramming characterized by reduced ribosomal protein synthesis, enhanced stress adaptation, and increased antibiotic tolerance [23, 37]. These findings reinforce the concept that tigecycline resistance results primarily from physiological adaptation mediated by transcriptional regulatory networks rather than structural modification of the ribosomal target.

**Fig. 1:**
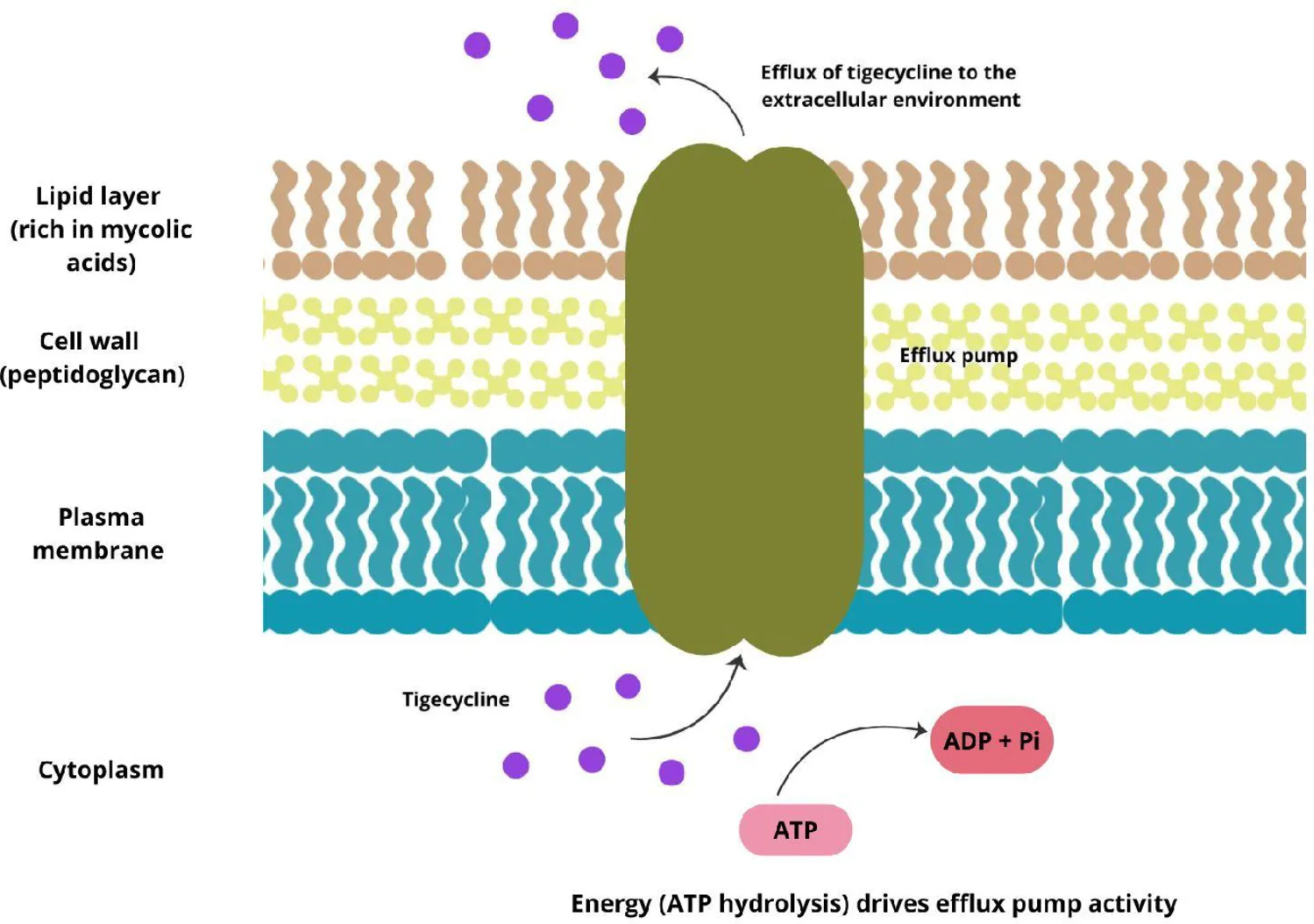
Efflux pump–mediated resistance to tigecycline in non-tuberculous mycobacteria (NTM). Tigecycline enters the bacterial cell via passive diffusion or porins, resulting in intracellular accumulation. Efflux pumps embedded in the plasma membrane actively recognize and export tigecycline to the extracellular environment. This process is driven by ATP hydrolysis (ATP → ADP + Pi), which provides the energy required for drug extrusion across the plasma membrane, peptidoglycan cell wall, and lipid-rich outer layer. Consequently, intracellular tigecycline concentrations are reduced, limiting binding to the 30 S ribosomal subunit and impairing inhibition of protein synthesis. This mechanism contributes to bacterial survival and promotes tolerance or resistance to tigecycline

Efflux pumps represent another major adaptive mechanism contributing to reduced intracellular tigecycline accumulation (Fig. 1). Members of the MmpL family, together with transporters belonging to the *ABCF *and Major Facilitator Superfamily (MFS), have been implicated in antimicrobial efflux in mycobacteria. Transcriptomic analyses indicate that exposure to tigecycline induces the expression of several efflux-associated genes in *M. abscessus*, reducing intracellular drug concentration and contributing to decreased susceptibility. Notably, the expression of many of these transport systems appears to be regulated directly or indirectly by WhiB7, highlighting the close interaction among the different resistance pathways.

Efflux pump inhibitors (EPIs) are a promising strategy to enhance the activity of tigecycline against NTM. Although no EPIs are specific to tigecycline, broad-spectrum efflux inhibitors have been used to investigate the contribution of efflux-mediated resistance to reduced tigecycline susceptibility. Among these, verapamil is the most extensively studied and has been shown to reduce the activity of multiple efflux pumps, potentially decreasing tigecycline minimum inhibitory concentration (MIC) values in some isolates [38, 39].

However, it is important to note that most of the available evidence regarding verapamil effect in NTM has been generated in combination with antimicrobials other than tigecycline. Experimental studies have demonstrated that verapamil enhances the activity of agents such as bedaquiline, clofazimine, rifampicin, and macrolides by inhibiting efflux systems and increasing intracellular drug accumulation. Therefore, the proposed benefit of verapamil in combination with tigecycline is based primarily on the recognized role of efflux pumps in modulating antimicrobial susceptibility rather than on studies specifically evaluating this combination [38].

Carbonyl cyanide *m*-chlorophenylhydrazone (CCCP) has also been evaluated as an efflux pump inhibitor [40]. Unlike direct efflux pump inhibitors, CCCP acts by dissipating the proton motive force, thereby impairing the energy source required for the activity of proton-dependent efflux pumps. This indirect mechanism can increase the intracellular concentration of tigecycline and reduce MIC values in some isolates, although the observed effects are variable [38].

Reserpine, in contrast, acts directly by inhibiting the activity of specific efflux pumps, particularly in members of the major facilitator superfamily (MFS). By blocking the transport function of these pumps, reserpine may also increase intracellular tigecycline accumulation and partially restore susceptibility in resistant isolates, although its activity appears to be strain-dependent and inconsistent across NTM species [38, 36].

However, as most evidence is still restricted to in vitro studies and the clinical application of EPIs remains experimental, their use currently represents a promising adjuvant strategy to enhance tigecycline efficacy and overcome one of the major resistance mechanisms in NTM [38]. Nevertheless, the absence of a consistent response to EPIs in some isolates suggests that reduced tigecycline susceptibility cannot always be explained solely by efflux activity and may also be associated with additional resistance mechanisms that remain poorly understood or have not yet been identified.

Other molecular determinants have been investigated but remain less well established. Ng et al. [28] initially identified mutations in *MAB_3542c* associated with tigecycline resistance; however, subsequent studies have not consistently reproduced these findings, limiting their acceptance as definitive resistance markers. Likewise, no consistent association has been demonstrated between mutations in *rpsA* and tigecycline resistance, further supporting the hypothesis that classical ribosomal target modification is not the predominant resistance mechanism in NTM.

Biofilm formation may also contribute indirectly to reduced tigecycline activity by limiting antibiotic penetration and promoting metabolically inactive bacterial populations with increased tolerance to antimicrobial exposure. However, direct evidence specifically linking biofilm formation to tigecycline resistance in NTM remains limited, and its precise contribution requires further investigation.

Taken together, these findings indicate that reduced susceptibility to tigecycline in NTM is not determined by a single molecular mechanism but rather by the coordinated activation of multiple adaptive pathways. Global transcriptional regulators, stress-response systems, and efflux mechanisms act synergistically to decrease intracellular drug activity, explaining the phenotypic variability observed among clinical isolates, particularly within the *M. abscessus* complex.

### Clinical implications and limitations

From a clinical perspective, the available evidence supports the continued use of tigecycline primarily for infections caused by rapidly growing nontuberculous mycobacteria (NTM), particularly the *M. abscessus* complex, in which it has consistently demonstrated lower MIC values compared to many alternative agents [33]. However, the increasing recognition of adaptive resistance mechanisms indicates that tigecycline should rarely be administered as monotherapy. Instead, combination regimens remain essential to minimize bacterial adaptation, enhance bactericidal activity, and reduce the likelihood of treatment failure [12]. In contrast, the consistent high MIC values observed in slow-growing species suggest that tigecycline has limited therapeutic applicability in these organisms [33].

These clinical considerations must be interpreted in light of important limitations in the current evidence. Most available studies are based predominantly on in vitro susceptibility testing, often involving relatively small collections of clinical isolates and focusing mainly on the *M. abscessus* complex. Comparative genomic analyses remain limited, and only a few studies have successfully correlated molecular resistance determinants with clinical outcomes. Additionally, substantial methodological variability in antimicrobial susceptibility testing, along with the absence of standardized clinical breakpoints for tigecycline, hampers direct comparison across studies and restricts the translation of MIC data into clinical decision-making.

In agreement with the evidence presented throughout this review, we consider tigecycline to exhibit favorable in vitro activity against clinically important NTM species and to have emerged as a promising therapeutic option, particularly for infections caused by rapidly growing mycobacteria such as *M. abscessus* and *M. fortuitum* [33]. Therefore, although tigecycline represents an important addition to the therapeutic arsenal against NTM infections, further clinical and microbiological studies remain essential to support its rational use and to improve treatment outcomes.

## Future directions

Future studies should prioritize multicenter investigations including larger and geographically diverse collections of clinical isolates, as well as the implementation of standardized susceptibility testing protocols. Integrated genomic and transcriptomic approaches will be essential to identify novel resistance determinants and to further elucidate the regulatory networks underlying adaptive resistance. Particular attention should be given to therapeutic strategies capable of overcoming inducible resistance mechanisms, including the evaluation of efflux pump inhibitors as potential adjuvants to conventional antimicrobial therapy. In addition, further research is needed to determine whether the resistance mechanisms described in *M. abscessus* are conserved across other NTM species, which may have important implications for broader treatment strategies. Finally, establishing robust correlations between in vitro susceptibility, molecular resistance mechanisms, and clinical outcomes will be critical for defining clinically relevant breakpoints and optimizing tigecycline-based therapeutic regimens.

## Supplementary Information

Below is the link to the electronic supplementary material.

Supplementary Material 1

## Data Availability

No datasets were generated or analysed during the current study.
