# Supplementary material for: A review of tigecycline resistance in nontuberculous mycobacteria
Source: Mol Biol Rep. 2026 Sep 27;53(1):1637. doi: 10.1007/s11033-026-12747-1 (PMC13616840; doi:10.1007/s11033-026-12747-1)
Supplement: Supplementary file 1 [file 11033_2026_12747_MOESM1_ESM.pdf]

Supplementary Table S1. The table shows search strategies and number of records identified in different databases before applying inclusion/exclusion criteria. Searches used combinations of terms for nontuberculous mycobacteria (NTM), tigecycline, and antimicrobial resistance.

**Data sources**	**Search strategy**	**Records identified before screening**	**Records after title/abstract screening**
Scopus	TITLE-ABS-KEY (Mycobacterium OR mycobacteria OR "nontuberculous mycobacteria" OR "non-tuberculous mycobacteria" OR NTM) AND TITLE-ABS-KEY (tigecycline OR tigeciclina) AND TITLE-ABS-KEY ("drug resistance" OR resistance OR "antimicrobial resistance" OR susceptibility OR "antimicrobial susceptibility" OR "minimum inhibitory concentration" OR MIC)	8	3
PubMed	(((Mycobacterium OR mycobacterium OR mycobacteria OR nontuberculous mycobacteria OR non-tuberculous mycobacteria OR NTM) AND (Tigecycline OR tigecycline) AND (Drug Resistance Bacterial OR resistance OR drug resistance OR antimicrobial resistance OR susceptibility OR antimicrobial susceptibility OR minimum inhibitory concentration OR	14	12

	MIC))) AND (y_5[Filter])		
SciELO	(Mycobacterium OR mycobacteria OR micobactéria OR micobactérias OR "nontuberculous mycobacteria" OR "non-tuberculous mycobacteria" OR micobactérias não tuberculosas OR MNT OR NTM) AND (tigecycline OR tigeciclina) AND (resistance OR resistência OR "drug resistance" OR "antimicrobial resistance" OR susceptibilidade OR susceptibility OR "minimum inhibitory concentration" OR "concentração inibitória mínima" OR MIC)	0	0
Web of Science	(TS = (Mycobacterium OR mycobacteria OR “nontuberculous mycobacteria” OR “non-tuberculous mycobacteria” OR NTM) AND TS = (tigecycline OR tigeciclina) AND TS = (“drug resistance” OR resistance OR “antimicrobial resistance” OR susceptibility OR “antimicrobial susceptibility” OR “minimum inhibitory concentration” OR MIC))	8	4
VHL	(((Mycobacterium OR micobacteria OR micobactéria OR "micobacterias no tuberculosas" OR	9	8

	NTM)) AND ((tigeciclina OR tigecycline)) AND ((resistência OR resistencia OR "drug resistance" OR suscetibilidade OR MIC)))		
LILACS	(((Mycobacterium OR micobacteria OR micobactéria OR "micobacterias no tuberculosas" OR NTM)) AND (tigeciclina OR tigecycline)) AND (resistência OR resistencia OR "drug resistance" OR suscetibilidade OR MIC)))	3	1
